# Transmucosal Absorption Enhancers in the Drug Delivery Field

**DOI:** 10.3390/pharmaceutics11070339

**Published:** 2019-07-15

**Authors:** Sam Maher, Luca Casettari, Lisbeth Illum

**Affiliations:** 1 School of Pharmacy, Royal College of Surgeons in Ireland, St. Stephens Green, Dublin 2, Ireland; 2 Department of Biomolecular Sciences, University of Urbino Carlo Bo, Piazza del Rinascimento 6, 61029 Urbino (PU), Italy; 3 IDentity, 19 Cavendish Crescent North, The Park, Nottingham NG7 1BA, UK

**Keywords:** permeation enhancers, absorption modifying excipients, oral delivery, nasal delivery, ocular delivery, vaginal delivery, transmucosal permeation

## Abstract

Drug delivery systems that safely and consistently improve transport of poorly absorbed compounds across epithelial barriers are highly sought within the drug delivery field. The use of chemical permeation enhancers is one of the simplest and widely tested approaches to improve transmucosal permeability via oral, nasal, buccal, ocular and pulmonary routes. To date, only a small number of permeation enhancers have progressed to clinical trials, and only one product that includes a permeation enhancer has reached the pharmaceutical market. This editorial is an introduction to the special issue entitled *Transmucosal Absorption Enhancers in the Drug Delivery Field* (https://www.mdpi.com/journal/pharmaceutics/special_issues/transmucosal_absorption_enhancers). The guest editors outline the scope of the issue, reflect on the results and the conclusions of the 19 articles published in the issue and provide an outlook on the use of permeation enhancers in the drug delivery field.

## 1. Introduction

Developing delivery strategies to assist movement of compounds across functionally conserved and structurally diverse epithelial barriers challenges drug delivery scientists. The most successful strategy to overcome poor permeation across epithelia, to date, has been to chemically modify the active compound to enable increased passive transport across non-injected epithelial routes. In cases where chemical modification is not feasible or unlikely to appreciably alter bioavailability, there may be no alternative but to formulate the active in an injectable dosage form, at considerable cost to manufacturers and inconvenience to patients. This is currently the case for many therapeutic macromolecules, including peptides, proteins and nucleic acids. Much of the early emphasis within the delivery field has focused on non-invasive administration of insulin; while it remains a major scientific challenge, oral delivery of other peptides, including GLP-1 receptor agonists may be preferable development candidates for Type II diabetes. Insulin is an affordable model peptide with established tools for qualitative and quantitative analysis, but there is interest in shifting towards development of smaller derivatised peptides [1] and cyclized peptides [2] that exhibit physicochemical properties that are more amenable to transmucosal permeation. Chemical engineering of poorly permeable macromolecules can improve potency, lengthen half-life, increase passive transcellular permeation and limit enzymatic degradation; these improved features may reduce the overall amount of active that is required to be shepherded across the epithelium. This may ultimately reduce the demand on the delivery system that is tasked with improving permeation across the epithelial barrier. Peptides and other macromolecules are not the only targets for assisted permeation across biological barriers. A percentage of marketed small molecule drugs, that exhibit low and variable bioavailability (Biopharmaceutics Classification System (BCS) Class III drugs), may show improved efficacy in second generation formulations containing delivery platforms. Additionally, successful delivery platforms may help to relax constraints on discovery screening of compounds that are predicted to exhibit low bioavailability, thereby enriching the pharmaceutical pipeline.

Inclusion of a chemical permeation enhancer (PE), sometimes referred to as an absorption modifying excipient (AME), in a dosage form is considered a simple approach to improve absorption across biological barriers. The most widely tested use of PEs today is in oral, nasal, pulmonary and buccal applications and to a lesser extent via rectal, ocular and vaginal routes. Altering the integrity of an epithelial barrier is met with caution within the field; especially as a wide range of potentially noxious compounds are included in PE categories (e.g., toxins [3]). Not all substances that alter barrier integrity can be considered candidate PEs, and it is only candidate PEs that are already used to improve delivery in marketed products that can be termed an AME. In reality however, only a small number of PEs have progressed to clinical testing, the majority of these having a history of safe use in humans (e.g., FDA-allowed excipients (ethylenediaminetetraacetic acid), to food additives (fatty acids), and endogenous secretions (e.g., bile acids)). Even in oral formulations that have advanced in clinical trials, the persistence of generally low and variable drug absorption hampers progress. Ongoing research attempts to address this problem through (i) identification of safer and more selective PEs that are not associated with some of the in vitro cytotoxicity that is observed with ionizable surfactants and chelating agents [4], and (ii) by investigating the impediments to translation of established PEs that have demonstrated promising enhancement action in pre-clinical delivery models.

This Special Issue presents an insight in to some of the current research on PEs. Research topics include assessment of natural, semi-synthetic and fully synthetic PEs in pulmonary, ocular, buccal, intestinal, vaginal and nasal delivery. Themes discussed in this issue include: bioassays to assess the effect of PEs on barrier integrity, identification of novel PEs, co-application of PEs with micro-and nano- encapsulation, comparison of mechanisms underpinning PEs that have been investigated in clinical trials, as well as a discussion of the technical challenges that continue to impede translation. Topics are broadly grouped as enteral (oral) or non-enteral (nasal, pulmonary and vaginal). As most PE applications pertain to oral and nasal routes of administration, reviews by Ghadiri et al. (nasal/pulmonary [5]), and Maher et al. (oral [6]) provide a current outlook on promising new developments by these major routes. Contributing authors also reviewed PEs by category [7].

## 2. Application of PEs via Enteral Routes

Delivery of peptides and proteins via the oral route is one of the key challenges in the delivery field. At present there are nine peptides administered by the oral route on the market, seven of which act locally, and only two are intended to reach systemic targets [8]. One of these two peptides is cyclised and lipophilic (cyclosporin) and so, only one conventional linear hydrophilic peptide (Desmopressin, 1069 Da) is marketed for systemic delivery via the oral route. Desmopressin is formulated in an immediate release tablet (0.2 mg, Desmotabs^®^, Ferring Pharmaceuticals, Saint-Prex, Switzerland), and has a bioavailability (BA) of only 0.16%. Additionally, concomitant ingestion with food decreases the extent of absorption by 40% [9]. The study of PEs in oral delivery is largely driven by the ambition to reformulate marketed injectables, but, unless an equivalent oral dosage form has a similar cost to the injectable formulation or has “extended value” over the injection (in terms of safety and/or efficacy), the patient may not be eligible for reimbursement, depending on the national healthcare system. In the absence of reimbursement for the oral tablet, patients may be unwilling to pay for the oral formulation, although there are exceptions (e.g., patients with needle phobia). Cost and reimbursement issues were several of the reasons behind the commercial failure of the first inhaled insulin (Exubera^®^) [10].

Evidence of enhancement of oral absorption of marketed injectable drugs provided a foundation for oral delivery of peptide analogues that exhibit high potency and long biological half-lives from the injected route (e.g., semaglutide with sodium salcaprozate (SNAC) [1] or stable insulin analogues with sodium caprate (C_10_)) [11]. That is not to say that some therapeutic peptides that are currently marketed in injectable and/or nasal formulations cannot be developed into commercially successful oral formulations. In 2015, the FDA agreed to accept the filing of a New Drug Application (NDA) for once daily oral salmon calcitonin (sCT) (TBRIA^TM^, Tarsa Therapeutics USA, Philadelphia, PA, USA). Additionally, Chiasma (Ness Ziona, Israel) is currently conducting a Phase 3 trial termed OPTIMAL (Octreotide capsules vs. Placebo Treatment In MultinationAL centres) for oral octreotide. In pre-clinical testing, several PEs categories have been extensively tested in oral delivery models, including surfactants, bile salts, chelating agents, toxins, and tight junction (TJ) modulators.

Maher et al. [6] provides a critical assessment of PEs in oral delivery. This review emphasizes the need to look beyond insulin to smaller peptides that may be more amenable to oral delivery, summarizes selected PEs in early development (e.g., choline geranate (CAGE) [12], permeant inhibitor of phosphatase (PIP) peptide 640 (PIP 640) [13]) and promising new formulations in the clinic [14], and explores some of the physiological challenges to translation of PEs in oral delivery. After a period of unsuccessful clinical trials and/or discontinuation of oral peptide programmes (e.g., salmon calcitonin (sCT) with Nordic Biosciences (Herlev, Denmark)/Novartis (Basel, Switzerland), there has been cause for optimism with positive clinical trial data emerging for selected peptide dosage forms, for example sCT with Tarsa Therapeutics (Philadelphia, PA, USA), octreotide with Chiasma and semaglutide with Novo Nordisk (Bagsværd, Denmark) [15], and ongoing effort to engineer stable peptides that are more amenable to oral delivery [2]. The lessons learned in unsuccessful clinical trials (reviewed in [16,17]) have also helped investigators identify the impediments to clinical translation, which has led to greater focus on formulation of PEs and efforts to translate promising pre-clinical data into an effective oral formulation that suitably co-presents the PE and the active in high concentrations at the intestinal epithelium. The article by Maher et al. also provides a commentary on the value of using simulated intestinal fluid in pre-clinical PE testing, an update on PE safety, and the lack of predictive power using certain animal models.

The review article by Twarog et al. [18] provides the first analysis of a head-to-head comparison between C_10_ and SNAC, two of the most widely tested PEs in oral delivery. Distinct mechanisms of action have been proposed for each based on in vitro data, although there remains uncertainty around their mechanisms at higher doses used in vivo. This article highlights the extensive history of clinical testing of both compounds, as well as the importance of both the selection of poorly permeable active and the design of the formulation. As things currently stand, SNAC has progressed further than C_10_, having gained approval for oral delivery of vitamin B_12_ as a medical food (Eligen^®^-Vitamin B_12_, Emisphere, Roseland, NJ, USA) and has completed Phase III studies in oral formulation of semaglutide with Novo Nordisk [19].

A number of novel PEs are discussed in this Issue, including tryptophan [20], lactose esters [21], sodium dilauramidoglutamide lysine [22] and ammonium mysristoyl chitosan [23]. Kamei et al. [20] performed a comprehensive study assessing the enhancement action of tryptophan, a hydrophobic amino acid that plays a key functional role in the action of cell penetrating peptides (CPPs) in pre-clinical delivery models. In rat ileal instillations, l-tryptophan improved bioavailability of insulin from 0.1% to 18.7% without causing mucosal injury. Other hydrophobic amino acids (isoleucine, proline, and phenylalanine) had no effect on insulin absorption. Further studies are required to elucidate the mechanism of enhancement action and to understand why this amino acid is effective in rat studies, but apparently not in Caco-2 monolayers. 

Lucarini et al. [21] performed a preliminary structure activity relationship on a panel of synthetic fatty acid lactose esters (hydrophobic chain lengths of C_10_, C_12_, C_14_ and C_16_). The authors recorded a decrease in the critical micelle concentration (CMC) for longer hydrophobic chain lengths, and an increase in cytotoxicity in Caco-2 and Calu-3 monolayers. At concentrations below the IC_50_ (half the maximum inhibitory concentration), there was no alteration to transepithelial electrical resistance (TEER) in Caco-2 monolayers, although there was a partial TEER reduction in lung epithelial cells; suggesting that Calu-3 cells may be more sensitive to the action of lactose esters [21]. 

Silva et al. [23] synthesized an amphiphilic quaternary ammonium chitosan derivative and assessed its capacity to improve solubility and in vitro permeability of the lipophilic small molecule, curcumin. Permeation of curcumin from micelles was assessed in Caco-2 monolayers and a tri-culture model (Caco-2, HT29 and Raji B cells). The study showed only a modest effect of the derivative on permeation enhancement, but the concept of using cationic amphiphilic agents for combined solubilization and permeation enhancement is worthy of further investigation. Additionally, this study highlights how mixed cell cultures can impact interpretation of how PEs alter barrier integrity. 

Haasbroek et al. [24] investigated how natural extracts from *Aloe vera* interact with intestinal epithelial cells. The study found whole leaf and gel extracts of *Aloe vera* contain considerable quantities of the acidifiers, citric acid and malic acid, which have previously demonstrated enhancement action [4]. Extracts caused a partial reduction in TEER and a two- to three- fold increase in permeation of fluorescein isothiocyanate (FITC) dextran 4 kDa (FD4) in Caco-2 monolayers. Confocal analysis of monolayers showed that FD4 was localized at the paracellular space, and that there was disruption of filamentous actin, a scaffolding protein that holds tight junctions in place. While this study does not provide definitive evidence of a paracellular effect over a transcellular effect, or elucidate the mechanistic steps leading to alteration in barrier integrity, the data shows that FD4 diffuses along the paracellular route. 

Bocsik et al. [25] performed a detailed evaluation of the interaction of the 18-mer CPP, PN159, with Caco-2 monolayers. This study highlights the difficulty of elucidating how PEs alter intestinal permeability. PN159 increased permeability and modulated the localization of TJ proteins. There was binding to claudins -4, and -7 at concentrations below a threshold for cytotoxicity in Caco-2 monolayers. Nevertheless, there was evidence of ultrastructural aberration and leakage of an extracellular dye into cells, suggesting a degree of transcellular perturbation. The authors therefore concluded that PN159 has a dual mode of action. It would be interesting to see if the paracellular mechanism can be uncoupled from cell penetrating effects via substitution of amino acid responsible for cell perturbation. Irrespective of the mode of action, PN159 caused a rapid reduction in TEER over 5 to 15 min that was partially recovered over 6 h and completely recovered after 24 h. There was a concomitant increase in permeability of fluorescent dextrans in the molecular weight range of 4–40 kDa.

Alama et al. [22] performed mode of action studies on SLG-30, a Gemini surfactant (so called for their distinctive chemical structure: hydrocarbon tail 1—ionic head group 1—spacer—ionic head group 2—hydrocarbon tail 2). In a head-to-head comparison, SLG-30 improved absorption of carboxyfluorescein in rat intestinal loop instillations by an order of magnitude more than two established PEs (sodium laurate and sodium glycocholate). Fluorescence anisotropy of brush border membrane vesicles showed that SLG-30 altered the membrane fluidity in the protein portion of enterocyte membrane as well as the inner leaflet of the plasma membrane. Concurrently, there was a significant reduction in the total cell expression of claudin-1 (57%) and claudin-4 (64%). These data emphasize how surfactant alteration to membrane architecture may directly influence TJ proteins and permeability via the paracellular route. 

A detailed understanding of how PEs alter permeability is an important aspect in PE selection, and there is often conflicting data within the scientific literature. Danielsen and Hansen [26] performed mode of action studies on two surfactants (sodium cholate and dodecylmaltoside (DDM)) in a jejunal mucosal explant [26]. This delivery model involved short-term culture of jejunal tissue segments in organ culture dishes with cell culture media. Sodium cholate and DDM caused leakage of Lucifer Yellow into epithelial cells as well as paracellular penetration of Texas Red dextran (3 kDa). At low concentrations (2 mM), there was no evidence of histological damage by light microscopy, although there was evidence of ultrastructural aberration by electron microscopy and histological damage was recorded at higher concentrations (10 mM). Both surfactants preferentially extracted non-lipid raft domains of the plasma membrane, suggesting these substances might cause perturbation at specific regions of the membrane rather than indiscriminate perturbation.

An extensive volume of literature has outlined the potential application of nanoparticles in a wide range of delivery applications. The initial premise that untargeted nanoparticles can facilitate significant translocation across the intestinal epithelium has not been forthcoming. There is demand to discover targeting ligands that can improve nanoparticle uptake such as through endogenous transporters on the mucosal surface (e.g., vitamin B_12_ [27]). Yong et al. [28] sought to determine if transferrin-mediated endocytosis can facilitate translocation of targeted polystyrene nanoparticles across Caco-2 monolayers. This study showed that polystyrene nanoparticles coated with an adsorbed layer of transferrin improved cellular uptake in non-polarized Caco-2 cells by 5-fold compared to the uncoated nanoparticles. In polarized Caco-2 monolayers, there was a 16-fold higher uptake of transferrin coated nanoparticles compared to non-polarized cells. There was also a 23-fold increase in permeation of transferrin coated nanoparticles compared to the non-coated particles. The authors conclude that the transferrin transport system may have potential application for both regional and systemic delivery of nanomedicines.

## 3. Application of PEs via Non-Enteral Routes

The nasal drug delivery technology market is expected to exceed $64 B by the year 2021 [29]. Nasal administration is among the most successful approaches for the systemic delivery of macromolecules. A number of small peptides are marketed in intranasal formulations, including; pritorelin (362 Da), oxytocin (1 kDa), desmopressin (1 kDa), buserelin (1.2 kDa), gonadorelin (1.2 kDa), nafarelin (1.4 kDa) and sCT (3.8 kDa) [30]. The largest of these peptides, sCT (Miacalcin^®^, Novartis, Switzerland), has a BA of approximately 1–3% with large variability (0.3–30%) [31]. None of these nasal peptide products contain a PE except for Miacalcin^®^, which contains benzalkonium chloride—a cationic surfactant that alters nasal permeability at selected concentrations [32]. PEs are commonly tested for nasal delivery of larger peptides and proteins (e.g., insulin, human growth hormone, interferon). Example PE categories for nasal delivery include; chitosan [31], non-ionic surfactants [33,34], CPPs [35,36], thiolated polymers [37], and cyclodextrins [38]. The non-ionic surfactants together represent the most clinically advanced PEs in nasal delivery (e.g., polyethylene glycol stearates and alkyl maltosides). DDM and tetradecyl maltoside (TDM) are constituents of Intravail^TM^ (Aegis, San Diego, USA acquired by Neurelis, San Diego, CA, USA), a delivery platform that has been approved for use to assist nasal absorption of sumatriptan (Tosymra^TM^, Dr Reddy’s, Hyderabad, India) [33]. In this Issue, Ghadiri et al. [5] discuss strategies for exploitation of PEs for improvement of intranasal and pulmonary delivery of macromolecules. PEs were grouped into five major categories; (i) surfactants, (ii) cyclodextrins, (iii) protease inhibitors, (iv) cationic polymers and (v) tight junction modulators. An overview of other approaches that may be used to improve permeability via the nasal and pulmonary routes is also provided. Additionally, promising PEs that have progressed to clinical testing are listed.

In their article, Pearson et al. continued the evaluation of the soluble non-ionic surfactant PE, polyethylene glycol (15)-hydroxystearate (Solutol^®^ HS15, recently re-branded as Kolliphor^®^ HS15, BASF, Ludwigshafen am Rhein, Germany) as part of the CriticalSorb^TM^ delivery platform (Critical Pharma, Nottingham, UK) [34,39]. This PE previously showed promising enhancement action in rats, where nasal BA of parathyroid hormone 1-34 (PTH1-34) was improved from 7.8% to 78% [40]. The current article examined intranasal administration of PTH1-34 in sheep (200 mcg) and humans (90 mcg) as either a liquid (containing 7.5% *w*/*v* Solutol^®^ HS15) or dry powder (40% *w*/*w* Solutol^®^ HS15). Overall, the promising PK data previously observed in rats were not replicated in large animals or humans. Nasal BA of PTH1-34 was 1.4% for the liquid and 1% for the dry powder in the ovine model. When the nasal spray was tested in seven healthy human volunteers, there were five non-responders. Mean BA was 0.26%, a value that increased to 1% when the non-responders were excluded. These values were considerably lower than the nasal BA of 3% observed in a previous Phase I study of human growth hormone (hGH) administered with Solutol^®^ HS15 in a dry powder formulation [41]. The nasal formulation caused mild irritation to the nasal cavity of sheep, but not in human subjects, which could be due to dose differences. Scintigraphy performed in humans showed the nasal spray was deposited in anterior segment of the nasal cavity, which may not be optimal for absorption of PTH1-34, and the authors acknowledge that BA may be improved if local residence time can be increased. Overall, further studies are necessary to determine the clinical potential of Solutol^®^ HS15 in nasal drug delivery.

Rassu et al. [42] assessed the effect of combining methyl-β-cyclodextrin (MβD) and chitosan chloride on nasal permeability of the model hydrophilic compound, N^6^-cyclohexyladenoside. Here, insufflation of spray-dried microparticles in rats improved BA in the order of [1:0] MβD:chitosan (BA: 36%) > [1:1] MβD:chitosan (BA: 12.8%) and [0:1] MβD:chitosan (BA: 1.85%) showing that the combination of the two PEs did not accentuate the enhancement action. The ADME data for these formulations compared favorably to an aqueous suspension of N^6^-cyclohexyladenoside administered via drops, which was below detectable levels in plasma of rats. There was also an undetectable level of N^6^-cyclohexyladenoside in cerebrospinal fluid. Interestingly, all test formulations containing PE also increased transport of N^6^-cyclohexyladenoside into cerebrospinal fluid, suggesting elevation in nose-to-brain transport.

Emulsomes are colloidal vesicular structures where a lipid core is coated with a phospholipid bilayer; thus, combining the properties of liposomes and simple emulsions. Additionally, excipients used in the preparation of emulsomes are often shown to alter epithelial permeability in pre-clinical animal models. El-Zaafarany et al. [43] describe the nasal delivery of oxcarbazepine loaded in emulsomes that were dispersed in a thermoresponsive gel. The emulsome in this formulation was composed of phosphatidylcholine: triolein (3:1) and polysorbate 80. There was comparable release of oxcarbazepine from the emulsomes and thermoresponsive gel over 8 h, but slower release from the gel over 24 h. In a previous study [44], intranasal administration of the emulsome thermogel to rats had a major impact on mean residence time (MRT) and oxcarbazepine area under the plasma concentration curve (AUC_0–48 h_) relative to either a suspension or solution dosage form. Additionally, there was a higher concentration of oxcarbazepine in the brain for a sustained period when administered in the combination of emulsomes with the thermoresponsive gel system.

Bento et al. [45] assessed a co-adjuvant strategy to improve nasal vaccine administration of recombinant hepatitis B surface antigen (HBsAg). The strategy involves combining a mast cell activator (C48/80) and a chitosan nanoparticle with adsorbed antigen, the rationale being that the immune potentiator activates the adaptive immune response while the nanoparticles improve immunogenicity of antigens. Loading efficiency for C48/80 chitosan nanoparticles (500 nm) was less than 20%, which was attributed to repulsion of cargo. There was considerable variation in the amount of model test antigen adsorbed to the particle surface (bovine serum albumen (BSA): ~90%, ovalbumin: ~70% and myoglobin: ~10%), possibly due to differences in the extent of protein ionisation. There was internalization of BSA-FITC in RAW 264.7 macrophages as measured by confocal microscopy. Intranasal administration of C48/80 loaded chitosan nanoparticles coated with HBsAg to C57BL/6 mice led to a higher anti-HBsAg IgG titer than another comparative nanoparticle (poly-ε-caprolactone [46]) by day 42. 

Bruinsmann et al. [47] developed a chitosan coated lipid-core nanocapsules for nose-to-brain delivery of simvastatin, a BCS Class II small molecule statin with potential neuroprotective actions [48]. The cationic lipid nanocapsules consisted of sorbitan monostearate and medium chain triglycerol surrounded by poly-ε-caprolactone and coated with low (21 kDa) or high (152 kDa) *Mw* chitosan. Mean particle size measurements for simvastatin loaded nanocapsules ranged between 163 to 168 nm for the low *M*w chitosan form and 161 to 210 nm for the high molecular weight forms. The higher *M*w nanocapsules had a slightly higher zeta potential (34 mV versus 29 mV in 10 mM NaCl), higher mucin weight ratios suggesting more efficient mucoadhesion and exhibited slightly slower release of simvastatin (31%) compared to the low *M*w (37%) and solution control (56%). There was an increase in simvastatin permeation in human nasal epithelial cells and isolated rabbit nasal mucosa with both chitosan nanocapsule prototypes. Permeation of simvastatin in rabbit nasal mucosa was 1.7-fold higher for the low *M*w chitosan capsules, which was attributed to differences in the physicochemical properties. Overall, more efficient mucoadhesion could improve localization of nanocapsules.

Ocular drug delivery can broadly be considered in terms of topical, peri-ocular, or intra-ocular administration. The most common route of administration is topical delivery where medicaments are directly applied to the cornea, sclera and conjunctiva for local or regional actions. Delivery to peri-ocular or intra-ocular targets via either the cornea or blood retinal barrier is difficult even for highly permeable actives. The cornea is a widely accessible epithelial surface, although drug transport across this barrier is arguably more challenging than via other epithelia. Aside from a relatively short residence time, there is likely to be legitimate concerns regarding the use of any additive that causes transmucosal perturbation. Moiseev et al. [49] comprehensively reviews the categories of PE (sometimes termed penetration enhancers via this route) assessed in ophthalmic delivery. This article includes an outline of ocular physiology and selected conditions where PEs may have potential application. A detailed description of major PE categories is provided: including cyclodextrins, chelating agents, crown ethers, CPPs, bile acids/salts, and a range of soluble and insoluble surfactants. The authors conclude that penetration enhancers act by modulating the tear film or epithelial integrity. While penetration enhancers may assist permeation of actives, it remains to seen whether the active can diffuse to intraocular or periocular targets for conditions such as age-related macular degeneration or diabetic retinopathy. 

While PEs primarily act to improve systemic absorption of poorly permeable actives, which can be achieved by enzyme inhibition, mucoadhesion and direct alteration to barrier integrity, these same functions may assist penetration of actives into tumors. Ci et al. [50] assessed the effect of a hydrogel containing cationic nanocrystals of imatinib/TPGS coated with polydopamine, to improve localization and penetration in a model of cervical cancer. The introduction of a cationic coating (polydopamine) inverted the zeta potential of imatinib nanocrystals from −9.27 mV to +27.25 mV and improved binding to mucin. The cationic charge enables mucoadhesion, and as cationic polymers have been shown to alter permeability, there is potential for improved transport with polydopamine. In the current study, the nanocrystals of imatinib had greater cellular uptake and improved anti-tumor efficacy in a murine orthotopic cervicovaginal cancer tumor model.

## 4. Review by PE Category

Promising PEs are also reviewed by category. The article by Peterson et al. is a useful guide to PEs from natural sources, which the authors collectively refer to as bioenhancers [7]. The article summarizes application of bioenhancers in buccal, nasal, oral and pulmonary routes of administration, and provides information on source, model of action, test delivery model and payloads. This comprehensive review discusses a wide range of phytochemicals that have not been previously reviewed in such detail, including *aloe vera*, black cumin, caraway, curcumin, diosmin and emodin.

## 5. An Outlook on PEs in the Delivery Field

PEs have been extensively tested via oral and nasal routes, and to a lesser extent via pulmonary, buccal, ocular and vaginal routes. As articles within this Issue highlight, there are challenges that impact translation of promising data observed in pre-clinical animal models into effective oral dosage forms in humans. The hurdle for investigators is to understand and control the action of PEs in dynamic environments observed in the GI tract, nasal cavity, lungs and oral cavity, preferably in animal models and humans. Co-localization of PE and active may help to replicate promising action that is observed in static delivery models. It may be that improving the effectiveness of PEs requires engineering technologies that co-localize the active and PE at the target epithelium. A focus on the final formulation requires convergence between formulation science, delivery science and engineering [51]. As academic research shifts from PEs towards nanoparticles and devices, there is less emphasis on addressing these hurdles to translation. This Issue also highlights that current emphasis in research on PEs remains on discovery of novel PEs that improve on potency, efficacy and safety of established candidates. However, improving these qualities may not negate issues related to co-delivery in dynamic environments. While discovery of new PEs that act via intricate and elegant paracellular mechanisms without evidence of mucosal perturbation is attractive, these PEs remain in preclinical research and have yet to be licensed to Pharma due to the higher risks: They have not improved upon the efficacy of leading PEs that have established safety records in humans. 

An additional highlight of this Issue is the evolving nature of the target drug. Non-invasive delivery of insulin was long considered the pinnacle of the drug delivery field, whereas today emphasis has switched to GLP-1 agonists and other peptides. This is not only because they are smaller and more amendable for transmucosal delivery, but also because they may have a higher therapeutic index. An oral insulin formulation may eventually progress, as major manufacturers report data on novel insulin analogues with improved stability in GI fluids and longer half-lives [11]. The importance of oral insulin is immediately recognized as a key challenge to the scientific community [52], but it is worth emphasizing there is unquestionable demand for practical, non-invasive delivery systems for other poorly permeable macromolecules, development of which could help to shape the future pharmaceutical pipeline with leads that are safer and more effective than conventional small molecules. As most marketed therapeutic peptides are small, stable and often cyclised, then it may be more pertinent to initially focus on developing prototype delivery systems and formulations for these target peptides. This may be difficult, as cost of more contemporary peptides and availability of suitable analytical methods can limit options available to academic investigators. 
